# Current Approaches in the Allocation of Liver Transplantation

**DOI:** 10.3390/jpm12101661

**Published:** 2022-10-06

**Authors:** Vlad Alexandru Ionescu, Camelia Cristina Diaconu, Simona Bungau, Viorel Jinga, Gina Gheorghe

**Affiliations:** 1 “Prof. Dr. Theodor Burghele” Clinical Hospital, University of Medicine and Pharmacy Carol Davila, 050474 Bucharest, Romania; 2 Department of Gastroenterology, Clinical Emergency Hospital of Bucharest, 105402 Bucharest, Romania; 3 Department of Internal Medicine, Clinical Emergency Hospital of Bucharest, 105402 Bucharest, Romania; 4 Medical Sciences Section, Academy of Romanian Scientists, 050085 Bucharest, Romania; 5 Department of Pharmacy, Faculty of Medicine and Pharmacy, University of Oradea, 410028 Oradea, Romania; 6 Department of Urology, “Prof. Dr. Theodor Burghele” Hospital, 050653 Bucharest, Romania

**Keywords:** liver transplantation, indications, contraindications, list management, donor base

## Abstract

In recent decades, important advances have been made in the field of liver transplantation. One of the major problems remaining in this area is the small number of donors. Thus, recent data bring multiple updates of the indications and contraindications of this therapeutic method. The main goal is to increase the number of patients who can benefit from liver transplantation, a therapeutic method that can improve life expectancy and the quality of life of patients with end-stage liver disease. Another goal in the management of these patients is represented by the optimal care of those on the waiting list during that period. A multidisciplinary team approach is necessary to obtain the best results for both the donor and the recipient.

## 1. Introduction

Over the past three decades, liver transplantation has become an option for saving the lives of patients with end-stage liver disease, liver malignancies, metabolic diseases with liver damage, or acute liver failure. The purpose of the transplant is to prolong the life of the recipient and improve their quality of life. According to data from the Organ Procurement and Transplantation Network, in 2012, more than 6000 liver transplants were performed in the United States (US) from brain-dead donors, while 240 transplants were performed from living donors [1]. A study published in 2018 reported a significant increase in the number of liver transplants (8082) performed in the US during that year [2]. However, the same study also highlights the large number of patients who remained on the waiting list, namely 13,869. Of these, 1169 died on the waiting list and 1304 were removed from the list because of worsening liver disease and failure to meet the criteria for transplant eligibility [2].

In Europe, the 2018 Liver Transplant Registry Report provides a comprehensive overview of the status and evolution of liver transplantation [3]. Thus, 146,782 transplants performed during 1968–2016 are mentioned from 169 centers in 32 European countries. To date, both the number of liver transplant centers and the annual number of liver transplants performed in Europe have gradually increased since the establishment of this registry date (7 liver transplants performed in Europe in 1968 vs. 5393 liver transplants in 2016) [3]. However, after an exponential increase in the 1980s, over the last few years, a plateau appears to have been reached, with an average of approximately 7700 liver transplants performed annually in Europe [3].

The outcomes for liver transplant patients continue to improve due to the optimization of both surgical techniques and immunosuppressive regimens and due to better management of infections and post-transplant complications.

Despite these successes, the spread of transplantation from brain-dead donors is limited by the reduced availability of donor organs [4]. A study assessing the evolution of liver transplantation in France, however, reports an increase in the rate of liver transplantation from brain-dead donors, from 23.8 cases per one million inhabitants in 2009 to 28.8 cases per one million inhabitants in 2017 [5]. The same study reports a family refusal rate for the years 2016–2017 of approximately 30–33% regarding organ transplantation [5]. 

The lack of donors remains, however, arguably the biggest challenge facing the liver transplant community today, with aggressive approaches to organ distribution and use being managed by an environment of intense government oversight [2].

## 2. World Liver Transplant History

The history of solid organ transplantation dates back several decades, but the success of this therapeutic option has been documented since the advent of more aggressive surgical techniques, immunosuppressive agents, and improved methods of organ preservation.

The earliest attempts at liver transplantation were in canine models but did not initially see therapeutic success. In 1952, Vittorio Staudacher, in Italy, published the first description of a canine liver transplant [6]. In the US in 1955, Stewart Welch described another canine transplant attempt, followed in 1956 by Jack Cannon of the University of California, Los Angeles, who described the concept of orthotopic transplantation [7].

These initial transplants led to the rapid death of the recipient but formed the basis for future studies. Shortly thereafter, in 1958, teams led by Thomas Starzl in Denver, Colorado, and Francis Moore in Boston, Massachusetts, performed canine liver transplants. They were technically successful but were doomed to failure due to poor organ preservation, as well as rapid organ rejection [6].

The first human liver transplant was performed by Thomas Starzl, Colorado, in 1963, the recipient being a pediatric patient with biliary atresia [8]. Unfortunately, the patient suffered intraoperative bleeding complications that led to death [8]. Over the next few years, there were other failed attempts by both Starzl’s team and other doctors. Finally, the first successful human liver transplant was performed by Starzl in 1967 on a 19-month-old patient with hepatoblastoma. The patient survived 13 months, later dying from a recurrence of the malignant tumor. This case was proof of the possibility of a successful liver transplant [8].

The acceptance of the concept of brain death in the US in 1968 was an additional milestone in the development of liver transplantation. The possibility of preserving the donor organs in physiological conditions led to a better quality and survival of the graft [9,10].

When the technical feasibility of liver transplantation was demonstrated, an important discovery was made in the field of immunological therapy. In 1969, cyclosporine was isolated from the Tolypocladiuminflatum fungus in Norway [11]. The introduction of cyclosporine in the late 1970s as part of the immunosuppressive regimen in organ transplantation improved the results through a lower toxicity and prevention of graft rejection, but also a lower rate of severe opportunistic infections compared to azathioprine [11]. In 1979, Calne first used cyclosporine in two patients who underwent liver transplantation [11,12]. Following the development and administration of cyclosporine, the medical and surgical outcomes of liver transplant patients improved. Later, the introduction of tacrolimus led to a further improvement in survival [13].

In June 1983, at the NIHCDC (National Institutes of Health Consensus Development Conference), after evaluating 531 cases, it was decided to approve liver transplantation as a valid therapy for the treatment of end-stage liver disease [12].

Since the 1990s, the field of liver transplantation has grown dramatically, with a huge expansion of the number of centers performing this procedure, and today there are hundreds of liver transplant centers in over 80 countries [9,14]. There has been a significant increase in the number of diseases associated with end-stage liver disease related to liver transplantation, and the contraindications have changed as the solutions to technical problems have improved. This has led to a shortage of donor organs, forcing transplant coordinators around the world to adopt organ allocation strategies [9,14]. In recent years, epidemiological data of patients undergoing liver transplantation, such as morbidity and mortality, have significantly improved. For example, a study published in 2018 reported a death rate of approximately 5.8% at one year after liver transplantation, and 11.3% at three years [15]. 

## 3. Liver Transplant Allocation

### 3.1. Indications for Liver Transplant

Liver transplantation is recommended in patients with end-stage liver disease, liver malignancies, liver metabolic disease, or acute liver failure (Table 1) [16,17,18,19,20].

### 3.2. Models of Prognosis and Organ Allocation

The natural history of cirrhosis is a continuum in which patients can progress from compensated liver disease to death. It is well known that decompensation due to complications of cirrhosis limits survival. This was demonstrated in a 2006 retrospective study comparing the results of two large studies in 1500 patients [21]. The authors observed that patients without portal hypertension had a much better evolution and prognosis compared to those with decompensated disease (average survival 12 years vs. 2 years) [21].

In view of these data, the authors suggested that cirrhosis should be divided into four “stages”, in which the initial stages (one and two) are considered compensated disease and the later stages (three and four) are considered decompensated disease [21].

Given the high rates of morbidity and mortality associated with end-stage liver disease, it is essential that physicians caring for such patients have a valid, reproducible system for staging disease severity and prognosis. Ideally, this system would be useful in determining those who need a transplant.

Traditionally, the two scores used in this area are the Child–Turcotte–Pugh (CTP) score and the model for end-stage liver disease (MELD) score, both of which are tools that help clinicians to assess the risk of mortality and the need for a transplant [22,23]. The CTP score was originally used as a prognostic tool to assess the risk of mortality in patients with liver disease who have undergone surgical treatment to alleviate portal hypertension [23]. The score includes: serum bilirubin, albumin, increased prothrombin time above control values, and the presence of hepatic encephalopathy and ascites [24]. One to three points are assigned for each degree of variation in the parameters and a composite score is obtained based on these (Table 2) [24]. The patient is included in a class according to the number of points: A (5–6 points), B (7–9 points), or C (10–15 points) [24]. An increase in CTP score suggests a low survival [24].

Prior to 2002, the CTP score was used as an index of disease severity for the allocation of livers from the brain-dead donor [23,24]. Patients were grouped (2A, 2B, or 3) based on CTP score (B vs. C), hospitalization status (outpatient, inpatient, or intensive care unit), and waiting time [25]. Thus, patients were sorted based on waiting time. Priority for transplantation was given to those who, despite having a less decompensated liver disease, had a longer waiting time [25,26]. In addition, the subjectivity of the variables of encephalopathy and ascites from the Child score led to differences in reproducibility between centers [25,26]. As a result, the transplant community sought a more objective index of disease severity and an allocation system that was not based on waiting time but on the severity of liver disease. The MELD score is a mathematical score based on three biochemical markers of liver function: serum bilirubin, INR, and creatinine [25,26]. It was originally used to predict survival after portosystemic transjugular shunt intervention (TIPS). As the liver function deteriorates, the MELD score increases, indicating an increase in the severity of liver disease [25,26]. Following several studies, the MELD score was validated as a tool for estimating the 90-day survival of patients with end-stage liver disease (including those on the transplant list) and was adopted in February 2002 by the United Network of Organ Sharing (UNOS) as the preferred system for organ allocation [25,26,27]. In the MELD era, those with the highest score have priority for transplantation and receive the organ first, regardless of factors such as the etiology of their liver disease or their waiting time. The use of the MELD score as a baseline score for organ allocation has led to decreased organ waiting times and increased transplant rates [25,26].

Despite its successes, there are disadvantages to the MELD allocation system [27,28]. There are clearly some patients who are disadvantaged by the MELD score [27,28,29,30]. These patients have diseases with an increased risk of mortality that cannot be expressed by INR, serum bilirubin, or serum creatinine. An example might be patients with severe portal hypertension, low MELD, rapid decompensation, or those with high mortality but less severe liver disease [28,29,30]. To address these issues, there have been suggestions to change the MELD score by introducing serum sodium into the equation (for refractory ascites or portal hypertension) or to take into account acute changes in the MELD score (MELD delta) to more accurately predict mortality and increase the priority for transplantation [29,30]. Hepatocellular carcinoma (HCC) is associated with a significant risk of mortality as a result of reduced hepatic synthesis function [27,28,29,30]. For this reason, patients with limited HCC are given “exceptional points” to accurately estimate the risk of mortality and to prioritize them for transplantation (Table 3) [31,32].

Since 1996, the Milan criteria (one nodule ≤ 5 cm or three nodules ≤ 3 cm, without vascular invasion) have been validated for liver transplantation in patients with HCC [33]. Applying these criteria, the recurrence of HCC after liver transplantation was below 10%. Milan citations are validated international criteria. There is an impetus from several teams to expand these criteria. In France, the Milan criteria have been replaced by the AFP score [34]. Therefore, some new criteria have been proposed:

- University of California in San Francisco (UCSF) criteria (1 nodule ≤ 6.5 cm, or n ≤ 3 nodules ≤ 4.5 cm, or total ≤ 8 cm) [35];
- Additions to the Milan criteria: number of nodules + maximum tumor size without vascular invasion should reach a maximum of seven [33];
- The AFP score, which takes into account the size, number of nodules, and level of AFP (applied in France) [34].

Due to improvements in the effectiveness of antiviral treatments, the number of patients with decompensated viral etiology (hepatitis B/C virus) is significantly decreasing [36]. In contrast, the number of patients with decompensated toxic-nutritional liver cirrhosis did not change, and the number of patients with decompensated liver disease due to non-alcoholic steatohepatitis increased, as some US studies have already shown [37].

Therefore, it is very important for organ distribution agencies to maintain a good balance between the different indications for liver transplantation, thus avoiding an increase in the mortality or abandonment of patients of any category on the waiting list. It is important to ensure access to liver transplantation for patients with decompensated liver disease, as well as for patients with HCC and those with an intermediate MELD score. Some authors believe that patients with MELD exceptions should have priority over those with very high MELD scores [31,32]. The perfect equation does not exist, but a real-time assessment of the dynamics of the waiting list by organ distribution agencies is essential to maintain fairness.

### 3.3. Pre-Transplant Evaluation

The purpose of liver transplant evaluation is to identify patients who will receive the greatest benefits from the transplant and who have the best chance of long-term survival. Additionally, the evaluation for transplantation serves to identify which complications of the liver disease can be modified, the conditions that can affect the result of the transplant, and the possible contraindications of the procedure. 

**Figure 1:**
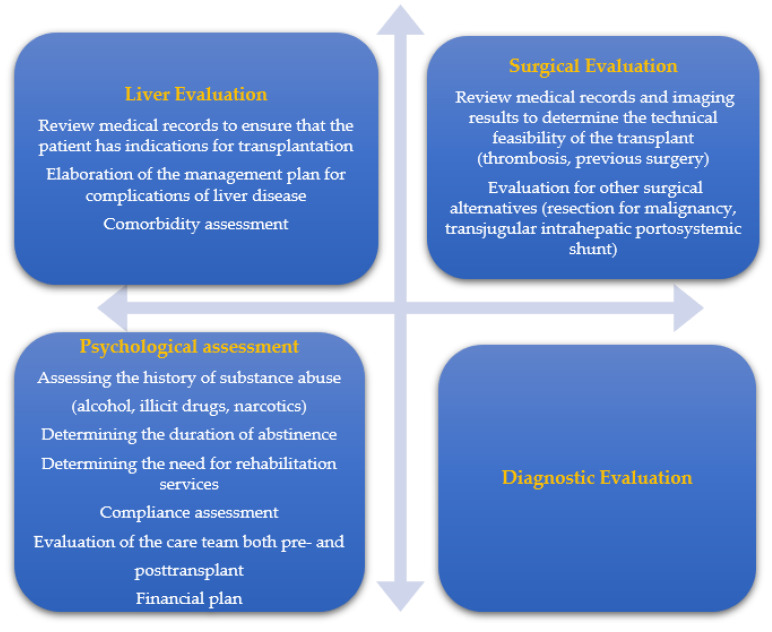
Multidisciplinary team needed for pre-transplant evaluation.

The diagnostic approach is multidisciplinary and involves evaluation at several levels: medical, psychiatric, social, and financial (Figure 1) [38,39,40,41,42,43,44,45,46]. Recent data support the important role of the evaluation of donor-specific antibodies (DSAs) for a proper allocation of the organs, to identify the patients at a high risk of graft loss and to establish individualized immunosuppressive regimens [47,48]. Legaz et al. highlighted a significantly lower survival rate among patients with a positive complement-dependent cytotoxicity crossmatch (CDC-CM) compared to patients with a negative CDC-CM (23.1% vs. 59.1%) [47]. The poor prognosis of patients with a positive CDC-CM was mainly due to the development of allograft rejection in the first 3 months [47].

Once the consultations and testing have been completed, a committee of regular gastroenterologists, transplant surgeons, coordinators, and psychiatrists is convened to select patients who meet the criteria for transplantation. When it is decided that a patient is a good candidate for transplantation, they are placed on the waiting list of the National Organ Distribution Network.

### 3.4. Absolute Contraindications for Liver Transplantation

In the process of choosing the right candidates, it is necessary to exclude those with extrahepatic diseases that confer a significant independent mortality risk (Table 4) [46,49,50]. For example, patients with severe heart disease, including pulmonary hypertension, or other severe comorbidities may be at an increased risk of intra-or postoperative death [46,49,50]. Transplantation is contraindicated in patients with uncontrolled or advanced hepatocellular carcinoma because recurrence rates are high [49].

Most US centers require patients with a previous non-hepatic malignancy to have a recurrence-free survival period before transplantation or a very low recurrence rate (i.e., high expectations of cancer cure) based on tumor type and stage [51,52]. Active and uncontrolled infections, particularly with fungi or resistant bacteria, at the time of transplantation are associated with a low survival [53]. Psychosocial contraindications are not always as well defined as medical ones, but they are just as important.

The transplant process is extremely complicated from evaluation to postoperative care; thus, patients who need a transplant must have an adequate social support system. Uncontrolled psychiatric disorders are contraindications to transplantation [54]. Due to the fact that many patients with liver disease have a history of substance abuse, strict abstinence from illicit drugs and alcohol is required before and after transplantation [55]. Some centers also call for an end to the use of narcotics and tobacco, but these are variable.

A lack of compliance with medical advice and indications are also contraindications to transplantation, as they predict an increased risk of non-compliance with post-transplant medical regimens [56].

### 3.5. Relative Contraindications for Liver Transplantation

The relative contraindications to transplantation are usually specific to each center and can sometimes be modified (Table 5) [46,49,50]. These contraindications increase the risk and complexity of surgery.

As life expectancy increases, the consideration of transplanting elderly patients is relevant in most US centers. There is no official age limit; however, the age of 70 years is considered the limit for transplantation due to comorbidities, perioperative mortality, and an increased risk of malignancy [57].

Many patients evaluated for transplantation are overweight or obese according to their body mass index (BMI). Studies on this subgroup have shown that mortality at 5 years was significantly higher in those with severe obesity and morbidity as a result of cardiovascular events [58]. Following these results, many centers have set a BMI value requirement for transplant listing [58].

Porto-pulmonary hypertension should be managed by an experienced team who can control high pulmonary pressures before, during, and after surgery [59,60]. Similarly, advanced hepatopulmonary syndrome with significant shunts and hypoxemia may be a relative contraindication to transplantation as it may require prolonged posttransplant mechanical ventilation [61].

Uncontrolled mental illness is an absolute contraindication to transplantation, and those with pre-existing mental illness should be under the care of a psychiatrist and have the disease well controlled before they can be accepted for transplant [62].

HIV infection was initially considered an absolute contraindication; however, improved treatment of HIV infection has made liver transplantation effective under certain conditions [63]. HIV patients should be referred to a transplant center with experience in managing specific issues related to HIV infection, both before and after transplantation [63].

### 3.6. Waiting List Management and Exclusion from the List

The success of liver transplantation has been considerable. Thus, in 2020, a total of 169,819 liver transplants (for the period 1968–2020) were reported in 32 countries and 171 centers [64]. This success can be explained by a very good survival rate after liver transplantation, 90% at 1 year and 80% at 5 years, respectively [65].

In Europe, most donors are brain-dead patients or patients who have suffered a cardio-respiratory arrest [66]. In these situations, the date of the transplant cannot be estimated, unlike the transplant from live donors, where the date is scheduled. Thus, waiting for a transplant may vary from a few days, in patients with acute hepatic impairment, to periods exceeding 1 year, for patients with cirrhosis of the liver or hepatocellular carcinoma [65].

Liver transplant waiting lists are mainly made up of three categories of patients:

- Patients with acute hepatic impairment, included in most countries on a “super-emergency” waiting list, giving them absolute priority over all other potential recipients. In this category of patients, the transplant should be performed within hours or days.
- Patients with decompensated liver cirrhosis. The priority of liver transplantation is dictated in this situation by the value of the MELD score. Thus, patients with a very high MELD score will be able to receive a liver transplant within days or weeks, while patients with an intermediate or low MELD score will wait for months or years.
- Patients with hepatocellular carcinoma or compensated liver cirrhosis. Organ transplant agencies give these patients an additional score depending on the size of the tumor. Generally, the transplant is performed within 18 months [67,68].

For patients with end-stage liver disease, the liver transplant procedure and the post-transplant period remain difficult. Therefore, these patients require prior training, both physical and mental. Managing patients on the waiting list is essential to avoid death or abandonment caused by the worsening of their health, but also to ensure the best possible physical condition prior to the surgical procedure. This is essential for postoperative success [65].

Once on the waiting list, patients should continue to be monitored for complications of end-stage liver disease. The transplant team should regularly evaluate patients for changes in their medical and mental condition, including:

- the usual evaluation of the MELD score;
- standard screening by upper gastrointestinal endoscopy for the presence of esophageal or gastric varices and their prophylaxis;
- in patients with hepatitis C-grafted hepatic cirrhosis, the opportunity for treatment with direct antiviral agents without interferon should be discussed [68,69];
- evaluation by abdominal ultrasound with vascular Doppler examination, every 6 months, to identify any tumors (hepatocellular carcinoma) or vascular complications;
- adequate prophylaxis of spontaneous bacterial peritonitis. Patients with refractory ascites may benefit from TIPS or repeated evacuation paracentesis.

In addition, physical activity during the waiting period has been shown to be beneficial for the patient and may improve VO_2_ [70,71].

When the patient’s condition has changed so that he or she can no longer survive a transplant or benefit from a transplant, the team may decide to remove a patient temporarily or permanently from the transplant list. Examples that could justify removal from the list are resistant bacterial or fungal infections, sepsis requiring vasopressors, the need for mechanical ventilation (especially with a high requirement for FiO_2_), or the need for hemodialysis [72].

In rare cases, removal from the transplant list may occur when the patient’s condition has improved such that the transplant is no longer justified [72].

Finally, given the organ deficit, liver transplantation should be performed in those patients who would benefit the most. It is also necessary to optimize the management of patients on the waiting list, to reduce the dropout rate and improve the results after liver transplant.

### 3.7. Allocation of Organs

When a potential organ donor is identified, the local organ procurement organization (OPO) is informed and begins to participate in the care of the donor organs. The OPO staff obtain a detailed medical history and perform tests for potentially communicable diseases. The recipient of that region or area with a compatible blood type and the largest medical emergency or the largest MELD is the first to be offered the organ of the donor. The transplant team has a limited amount of time to consider the information obtained about the donor and the likelihood of an appropriate match between the organ and the recipient [73,74,75,76].

If the transplant team accepts the organ, a sampling team is required to harvest the organ in a timely manner. If the transplant team refuses the organ, it is offered to the next candidate on the waiting list until it is accepted [73,74,75,76].

### 3.8. Extension of the Donor Base

**Figure 2:**
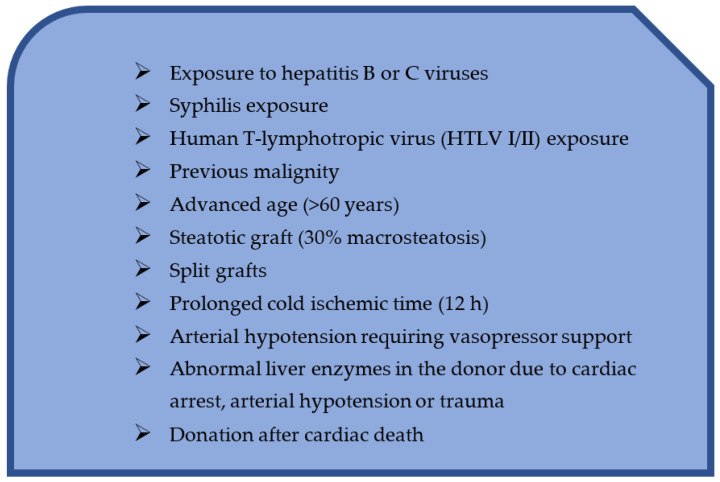
Criteria for expanding the donor base.

While liver transplantation results continue to improve, this intervention is limited by the low availability of donor organs. Every year, thousands of potential recipients die while on the waiting list due to the inadequate organ supply. Out of a desire to improve this, many centers encourage patients to follow up on living donors or to extend the criteria for organ donation (Figure 2) [77]. 

Extensive criteria for organ donors are those that pose a certain additional risk to the recipient, such as the risk of disease transmission or the risk of low initial or long-term function [77].

These organs usually come from older donors, donors with a history of exposure to viral hepatitis or other infection, previously cured malignancies, a history of high-risk social behavior which is considered to be a risk factor for communicable diseases, partial grafts, or organs donated after cardiac death [77,78,79].

Most organs are from brain-dead donors. These are donors who have suffered irreversible neurological damage with a loss of cortex and brainstem function. Those who have suffered serious neurological damage or other critical illnesses without a reasonable chance of a significant recovery but who do not meet the criteria for brain death may still be considered donors after a sudden cardiac death, with the consent of the family. This type of donor is called a cardiac death donation (DCD) [80]. Some centers report acceptable transplant results with DCD grafts when the ischemia time is kept to a minimum. These reports have led to a slight increase in the use of DCD organs, despite concerns about the high rate of primary graft dysfunction, biliary ischemia, and the need for re-transplantation due to prolonged graft ischemia [80].

The regenerative capacity of the liver allows the donor base to be expanded by two mechanisms—division of the transplanted liver and liver transplantation from a living donor [81]. Partial or dissociated liver cells allografts allow the division of a single organ from the deceased donor to two recipients [82]. Divided allografts are usually divided between a pediatric patient (left hepatic lobe or left lateral lobe) and an adult patient (right lobe) [82]. Reports suggest that graft insufficiency after split liver transplantation is only increased when these grafts are used in patients with fulminant liver failure and malignancy, suggesting that—with the exception of these two diagnoses—this method can be used [83,84]. The success of split liver transplantation has led to the emergence of liver transplantation from living donors, becoming an important avenue for recipients to benefit from liver transplants. Particular attention is being paid to the assessment of potential living donors to ensure adequate social support but also in assessing health and anatomy [83,84]. 

Although there are many benefits of transplanting from living donors, the most important are providing much earlier access to a recipient organ and preventing the progression to a complication of liver disease or even death [85]. In addition, a transplant from a living donor removes the recipient from the waiting list, allows for a transplant with a short ischemic time, and provides an even healthier organ if the donor is well evaluated [85]. 

The results of those who have received a liver from a living donor are similar or even slightly improved compared to those who have received a liver from a deceased donor. One study showed that adults who received a liver from a living donor had a lower mortality rate than those who remained on the waiting list for liver transplant [86]. The outcomes of patients who received transplants from living donors depend to a large extent on the experience of the center; the centers that performed more than 20 transplants obtained better results and lower post-transplant complication rates [86,87].

Although transplantation from a living donor is associated with excellent results, the use of this technique is somewhat limited, as it requires the presence of a distinct donor team. As with potential recipients, potential donors must go through an intensive medical, surgical, and psychological assessment, an assessment of anatomical compatibility, and must demonstrate a clear understanding of the risks associated with the procedure. The procedure requires two qualified surgical teams working together to achieve excellent results for both the donor and the recipient. The risks for donors, although small, include infection, strictures of the bile ducts, the need for transplantation, and death—the latter two being rare (<1%) [85,86,87]. 

## 4. Conclusions

Liver transplantation is a therapeutic option for patients with end-stage liver disease. To obtain the best results for both the donor and the recipient, a key element is the optimal management of organ allocation. The whole process—from being placed on the waiting list to receiving a liver for transplant—is complex, involving a multidisciplinary team. In recent decades, considerable progress has been made in the field of liver transplantation, where the minimum goal is represented by an increase in the number of patients who can benefit from this therapeutic method.

## Figures and Tables

**Table 1:** Indications for liver transplantation.

Indications for Liver Transplantation.
➢ Viral hepatitis - Hepatitis B - Hepatitis C ➢ Autoimmune liver disease ➢ Toxic liver disease ➢ Metabolic liver disease: - Hereditary hemochromatosis - A-1-antitrypsin deficiency - Wilson’s disease - Tirozinemia - Non-alcoholic hepatic steatosis - Glycogen storage disease type IV - Amyloidosis - Neonatal hemochromatosis - Hyperoxaluria - Urea cycle disorders - Amino acid metabolism disorders ➢ Cholestatic liver disease: - Primitive biliary cirrhosis - Primitive sclerosing cholangitis - Biliary atresia - Alagille syndrome - Progressive familial intrahepatic cholestasis - Cystic fibrosis ➢ Malignancies: - Hepatocellular carcinoma - Cholangiocarcinoma - Fibrolamellar hepatocellular carcinoma - Hepatoblastoma - Metastases of neuroendocrine tumors ➢ Polycystic liver disease ➢ Vascular disease: Budd–Chiari syndrome Acute fulminant liver failure

**Table 2:** CTP score and 1 and 2-year survival rates.

Points	1	2	3
Hepatic encephalopathy	None	Minimal	Advanced
Ascites	None	Slight	Moderate
Serum bilirubin (mg/dL)	<2.0	2–3	>3.0
Serum albumin (g/dL)	>3.5	2.8–3.5	<2.8
Prothrombin time (seconds)	1–4	5–6	>6
CTP classes		Score	
A		5-6	
B		7-9	
C		10-15	
Survival		1 year	2 years
A		100%	85%
B		80%	60%
C		45%	35%

**Table 3:** Exceptional MELD score points appraisal conditions.

Exceptional MELD Score Points Appraisal Conditions
Hepatocellular carcinoma Cholangiocarcinoma Familial amyloidosis Primary hyperoxaluria Hepatopulmonary syndrome Porto-pulmonary hypertension Refractory pruritus Refractory ascites Budd–Chiari syndrome Symptomatic polycystic liver disease Hereditary hemorrhagic telangiectasia Polycystic liver disease Recurrent bacterial cholangitis Recurrent variceal bleed Other gastrointestinal bleeding (not due to varices) Spontaneous bacterial peritonitis Hepatic encephalopathy

**Table 4:** Absolute contraindications for liver transplantation.

Absolute Contraindications
Severe and irreversible comorbidities with a negative impact on long-term life expectancy short Severe pulmonary hypertension (mean pulmonary arterial pressure ≥ 50 mmHg) Extrahepatic malignancies (except for certain forms of skin cancer) Extensive hepatocellular carcinoma or macrovascular or lymph node invasion Cholangiocarcinoma Uncontrolled sepsis Inadequate social support Alcohol and/or drug abuse Unacceptable risk of recurrence Severe uncontrolled psychiatric illness

**Table 5:** Relative contraindications for liver transplantation.

Relative Contraindications
Advanced age (>70 years) Moderate pulmonary hypertension (mean pulmonary blood pressure between 35 and 50 mmHg) Severe hepatopulmonary syndrome with PaO_2_ ≤ 50 mmHg Morbid obesity (body mass index ≥ 40 kg/m^2^) Extensive portal thrombosis and mesenteric thrombosis MELD score < 15 HIV infection Reduced compliance Psychiatric comorbidities

## Data Availability

Not applicable.
